# Effectiveness and cost-effectiveness of early assisted discharge for Chronic Obstructive Pulmonary Disease exacerbations: the design of a randomised controlled trial

**DOI:** 10.1186/1471-2458-10-618

**Published:** 2010-10-18

**Authors:** Cecile MA Utens, Lucas MA Goossens, Frank WJM Smeenk, Onno CP van Schayck, Walter van Litsenburg, Annet Janssen, Monique van Vliet, Wiel Seezink, Dirk RAJ Demunck, Brigitte van de Pas, Peter J de Bruijn, Anouschka van der Pouw, Jeroen MAM Retera, Petra de Laat-Bierings, Loes van Eijsden, Maria Braken, Riet Eijsermans, Maureen PMH Rutten-van Mölken

**Affiliations:** 1 Department of Respiratory Medicine, Catharina Hospital Eindhoven, Eindhoven, the Netherlands; 2 Institute for Medical Technology Assessment, Erasmus University, Rotterdam, the Netherlands; 3 Department of General Practice, School for Public Health and Primary Care (CAPHRI), Maastricht University, Maastricht, the Netherlands; 4 Department of Respiratory Medicine, Atrium Medical Centre, Heerlen, the Netherlands; 5 Department of Respiratory Medicine, Máxima Medical Centre, Veldhoven/Eindhoven, the Netherlands; 6 Department of Respiratory Medicine, Alysis zorggroep Rijnstate Arnhem, Arnhem, the Netherlands; 7 Department of Respiratory Medicine, TweeSteden Hospital, Tilburg, the Netherlands; 8 Department of Health Care Policy, Meander Group Zuid-Limburg, Heerlen, the Netherlands; 9 Department of Staff Nurses Nursing and Care, ZuidZorg, Veldhoven, the Netherlands; 10 Department of Transmural Care, Thebe, Tilburg, the Netherlands

## Abstract

**Background:**

Exacerbations of Chronic Obstructive Pulmonary Disease (COPD) are the main cause for hospitalisation. These hospitalisations result in a high pressure on hospital beds and high health care costs. Because of the increasing prevalence of COPD this will only become worse. Hospital at home is one of the alternatives that has been proved to be a safe alternative for hospitalisation in COPD. Most schemes are early assisted discharge schemes with specialised respiratory nurses providing care at home. Whether this type of service is cost-effective depends on the setting in which it is delivered and the way in which it is organised.

**Methods/Design:**

GO AHEAD (Assessment Of Going Home under Early Assisted Discharge) is a 3-months, randomised controlled, multi-centre clinical trial. Patients admitted to hospital for a COPD exacerbation are either discharged on the fourth day of admission and further treated at home, or receive usual inpatient hospital care. Home treatment is supervised by general nurses. Primary outcome is the effectiveness and cost effectiveness of an early assisted discharge intervention in comparison with usual inpatient hospital care for patients hospitalised with a COPD exacerbation. Secondary outcomes include effects on quality of life, primary informal caregiver burden and patient and primary caregiver satisfaction. Additionally, a discrete choice experiment is performed to provide insight in patient and informal caregiver preferences for different treatment characteristics. Measurements are performed on the first day of admission and 3 days, 7 days, 1 month and 3 months thereafter. Ethical approval has been obtained and the study has been registered.

**Discussion:**

This article describes the study protocol of the GO AHEAD study. Early assisted discharge could be an effective and cost-effective method to reduce length of hospital stay in the Netherlands which is beneficial for patients and society. If effectiveness and cost-effectiveness can be proven, implementation in the Dutch health care system should be considered.

**Trial registration:**

Netherlands Trial Register NTR1129.

## Background

Chronic Obstructive Pulmonary Disease (COPD) is a chronic disease that is currently ranked as fourth major cause of death globally [1]. Due to an aging population and late effects of smoking, the prevalence of COPD will increase in the following 20 years [2]. Projections for the year 2030 indicate that COPD will be third major cause of death, as a result of the projected increase of tobacco use, especially among women and low-and middle income countries [1]. COPD is characterised by an airflow limitation which is not fully reversible. Symptoms include sputum production, cough and dyspnoea. These symptoms are chronic and progressive over time [3].

Acute exacerbations of COPD can be defined as 'an event in the natural course of the disease characterized by a change in a patients' baseline dyspnoea, cough and/or sputum production that is beyond the day-to-day variations, is acute in onset, and may warrant a change in regular medication in a patient with underlying COPD' [3]. The exacerbation frequency is dependent on several factors including disease severity and number of exacerbations in the previous year [4,5]. Although most exacerbations are treated in the community [5], exacerbations are the main cause for hospitalisations in COPD patients [5,6]. Studies have shown that exacerbations and hospital admissions negatively influence patient outcomes, by increasing lung function decline [5,6], decreasing quality of life [7,8], increasing mortality [9] and increasing readmission risk [8,10].

With a mean length of stay in the hospital of 9 days [6,11] the large number of hospital admissions for exacerbations among COPD patients result in a high pressure on scarce hospital beds and high health care costs, accounting for up to 70% of total expenses for COPD [12,13]. Even without intervention, hospital costs will rise as a result of the increasing prevalence of COPD, especially among women. To reduce health care costs, alternatives for hospital treatment have been developed. One alternative that has gained popularity in the last 15 years are hospital at home schemes [14,15]. These schemes aim at reducing the pressure on hospital beds and overall health care costs without negatively influencing the patient outcomes and increase patient satisfaction [16].

### Hospital at home and early assisted discharge

Hospital at home is defined as "a service that provides active treatment by health care professionals in the patient's home for a condition that otherwise would require acute in-patient care, and always for a limited time period"[17]. Hospital at home is also known as 'home hospitalisation' or 'hospital in the home'. Depending on the target population of the scheme and the type of care provided, schemes vary in organisational structure and may involve different professionals [17]. Hospital at home schemes can be divided in admission avoidance schemes, (early) assisted or supported discharge schemes and combined schemes. Depending on who bears the financial and management responsibilities, the schemes can further be divided in community resourced or hospital resourced. Community resourced schemes commonly built on the existing infrastructure for care provision in the community, whereas hospital resourced schemes work on an outreach basis and home care is provided by hospital staff.

### Hospital at home for COPD exacerbations

Hospital at home schemes for the treatment of COPD exacerbations specifically, have been studied in several randomised controlled trials [18-25] and various non-randomised studies including observational studies [26-31] and studies with retrospective analysis [32]. Studies were performed in the United Kingdom [19,20,24-29,32], Ireland [30], Australia [23], Italy [18] and Spain [21,22,31]. These studies showed that approximately 25% of all patients with an acute exacerbation of COPD can be treated at home safely with no negative effects on their health outcomes and with great patient satisfaction [16]. These results triggered the wide implementation of hospital at home schemes for COPD exacerbations in the United Kingdom over the last 10 years [14,15]. In 2007, the British Thoracic Society developed the Hospital-at-Home in Chronic Obstructive Pulmonary Disease guideline providing a framework for the development and adjustment of hospital at home schemes [33].

Most hospital at home schemes active in the United Kingdom are assisted discharge schemes, with specialised respiratory nurses providing home care on an outpatient basis [14,15]. However, it remains unknown whether this is the most effective model for hospital at home care. The use of generic community district nurses or telephone monitoring might be an option that increases the capacity of the hospital at home schemes for COPD exacerbation. Supported by their positive results, Davison et al. [28] suggest that the use of generic community nurses in hospital at home schemes should be studied more intensively.

Hospital at home for COPD exacerbations initially requiring hospital admission has also been the subject of several cost or cost-effectiveness studies [18,22,23,25,34-36]. Significant and substantial cost savings were found in Australia (€1200 per episode) [23], Spain (€800) [22,35] and the United States (€1700) [37]. No significant cost savings were found for England and Italy [36]. (Amounts were converted to Euros, using exchange rates of March 2010). All studies were performed from a health care or payer perspective, meaning that they recorded only costs in the health care sector and not included costs of informal care. Although treating COPD exacerbations at home has the potential to reduce costs, whether and the extent to which it does so in the Netherlands is unknown. Apart from the exact organisation of the hospital at home scheme, its health economic impact depends heavily on national and local treatment patterns, health care delivery structures, funding and reimbursement systems, absolute and relative differences in unit costs of resource use and drug prices. The limited transferability of cost-effectiveness results to other settings stresses the need for setting-specific cost-effectiveness studies.

This contribution presents the design of the GO AHEAD trial (GO AHEAD is an acronym for Assessment Of Going Home under Early Assisted Discharge). In this Randomised Controlled Trial (RCT) patients admitted to the hospital for an exacerbation of their COPD are discharged early and monitored at home by nurses.

### Research questions

Our primary research question is: "What is the effectiveness and cost-effectiveness of an early assisted discharge intervention compared to hospital care as usual for patients hospitalised with an exacerbation of their COPD." The primary measure of effectiveness will expressed by the change in health status, measured by the change in Clinical COPD Questionnaire (CCQ) [38] scores between randomisation and day 7, while costs include COPD-related health care costs, patients' and informal caregivers' out-of-pocket costs and patients' and informal caregivers' costs of production loss.

The following secondary research questions will be addressed:

1) What is the long-term effectiveness of early assisted discharge compared to hospital care as usual?

2) What is the difference in treatment failures between the early assisted discharge scheme and usual care in hospital?

Treatment failure in the intervention group is defined as readmission before day 7 or death before day 7. In the control group treatment failure is defined as death before day 7 or clinical deterioration leading to prolongation of hospital stay after day 7.

3) What is the effect of early assisted discharge on readmission rates after discharge from hospital or the early assisted discharge scheme, in comparison with usual care in hospital?

4) What is the effect of early assisted discharge on mortality after discharge from hospital or early assisted discharge scheme, in comparison with usual care in hospital?

5) What is the effect of early assisted discharge on patients quality of life in comparison with usual care in hospital?

6) What is the effect of early assisted discharge on primary informal caregiver burden in comparison with usual care in hospital?

7) How is patient and primary informal caregiver satisfaction with the early assisted discharge scheme compared with usual care in hospital?

Additionally a discrete choice experiment (DCE) is performed in order to provide more insight in patient and primary informal caregiver preferences for different treatment characteristics.

## Methods/Design

### Study design

**Figure 1:**
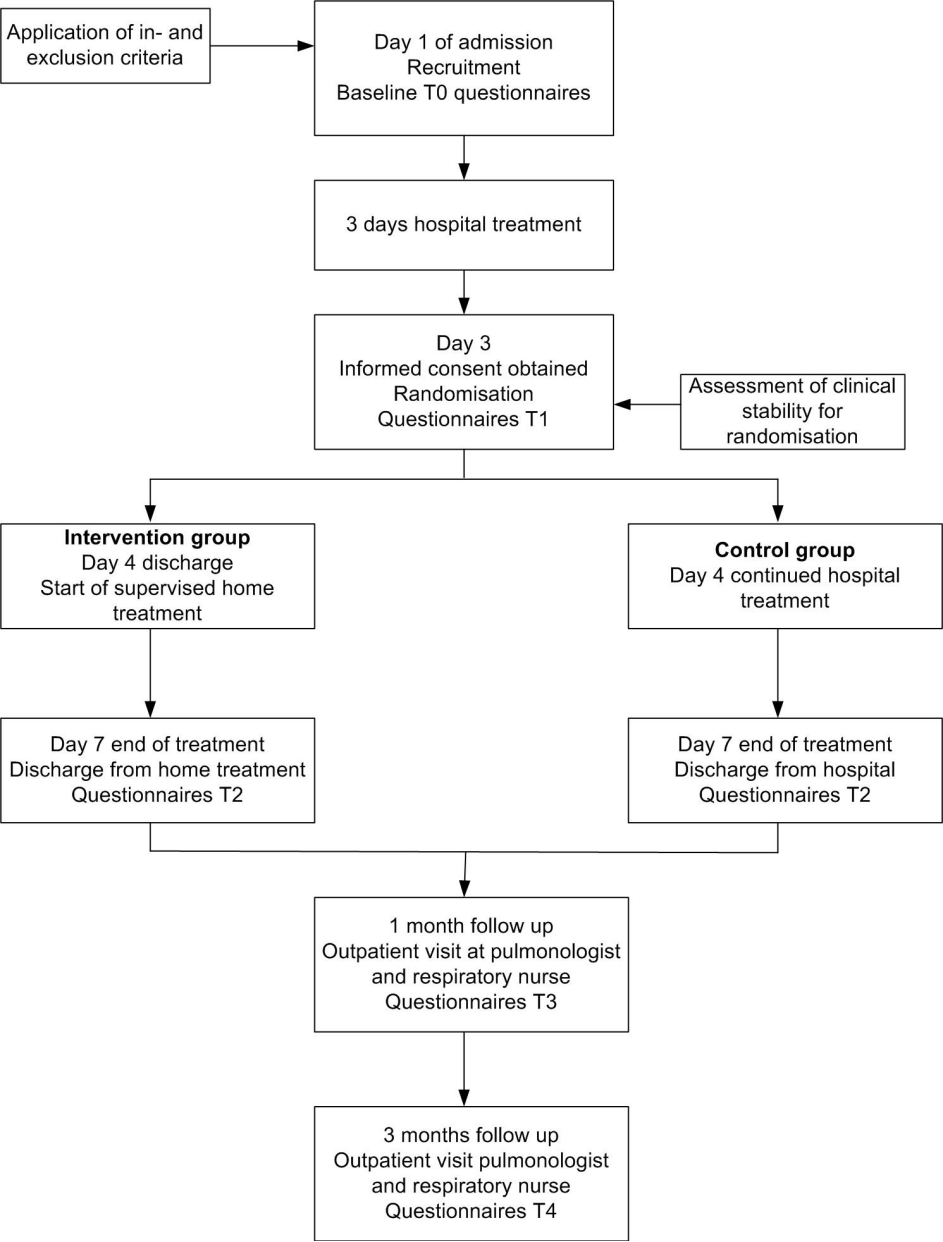
**Study design**.

The GO AHEAD study is a randomised controlled, multi-centre trial comparing two management strategies for patients admitted to the hospital for a COPD exacerbation. The intervention strategy is early assisted discharge, which implies that patients are discharged early from hospital with a package of home care. Recovery is monitored while patients are further treated at home. This management strategy is compared to usual hospital care, where patients remain hospitalised and are monitored in hospital. The total length of the active, supervised treatment phase for both groups is planned to be seven days. The follow up period of the trial is three months. Figure 1 gives a complete overview of the study design. Main focus of the study is not only to perform an effect evaluation, but also a cost evaluation and a discrete choice experiment. This trial was approved by the Medical Ethics Committee of the Catharina-hospital Eindhoven, the Netherlands and this approval was reconfirmed by the Medical Ethics Committees of the other participating hospitals.

### Setting and recruitment

Patients admitted to one of the participating hospitals because of an exacerbation of their COPD, through either the emergency and accident department or after an unscheduled outpatient visit to the pulmonologist, are screened for eligibility. Patients are assessed for eligibility at two time points. On day one, the pulmonologist and research nurse screen the patient for eligibility according to the inclusion and exclusion criteria listed in table 1. On day 1 patients are considered eligible for potential early discharge if they meet the following inclusion criteria: aged 40 or over, competent, diagnosed with at least COPD GOLD stage 1 (post-bronchodilator FEV_1_/FVC < 70% [3]) and a smoking history of minimally 10 pack years (PY), hospitalised with a moderate to severe exacerbation and finally, completed informed consent on day three of admission. Patients are excluded if they meet any of the following exclusion criteria: major uncontrolled comorbidity, mental disability, active alcohol- or drug abuse, inability to understand the program, living outside the region of the participating home care organisation, indication for admission to the intensive care unit or non-invasive ventilation and insufficient availability of informal care at home.

**Table 1:** Inclusion and exclusion criteria

Inclusion criteria:
Age 40 years and over

Competent

Diagnosed with at least COPD GOLD I and 10 Pack Years of smoking or grounded susceptibility for COPD

Hospitalised with COPD exacerbation

Completed informed consent on day 3 of admission



**Exclusion criteria:**

Major uncontrolled comorbidity

Mental disability

Active alcohol abuse and/or drug abuse

Inability to understand the program

Living outside care region of the home care organisation

Indication for admission to intensive care unit or non invasive ventilation

Insufficient availability of informal care at home

On day 1, all patients considered eligible for potential early discharge are invited to participate in the controlled clinical trial in which they either continue their hospital admission or are discharged early to home care. These patients are informed that, if they fulfil the in- and exclusion criteria and are still willing to participate on day 3, they will be randomised on day 3 and discharged on day 4. For patients admitted before 12:00 pm, the day of admission is considered as day 1, otherwise, the following day is considered as day 1.

Patients must have signed the informed consent form before randomisation on day 3, which means they have three days to decide whether they want to participate in the trial or not. This procedure is possible because the treatment in the first three days is not different from that of patients not participating in the trial. Data collected before the third day of admission will be destroyed if the patient does not give informed consent on that day. This procedure has been approved by the ethics committee.

Patients who refuse to participate in the trial are invited to participate only in the discrete choice experiment study without participating in the RCT. These patients are contacted by telephone one month after admission and asked to give informed consent for this part of the study. This informed consent form is different from the form that is used in the RCT.

### Randomisation procedure

On the third day of admission clinical stability is assessed in order to determine whether patients can be randomised. This design for randomisation is adapted from the Spanish study performed by Diaz et al. [21] although the criteria for clinical stability were adapted to represent the current practice in the participating hospitals and because of the difference in treatment package patients receive at home. Patients need to meet the following criteria to be randomised: 1 acceptable general health defined as decrease of physical complaints, non dependency of therapies that can not be given at home and being able to visit the toilet independently; 2 normal or moderately increased blood sugar levels, defined as ≤ 15 mmol/L or ≥ 15 mmol/L while the patient is capable to regulate blood sugars independently at home; and respiratory complaints of dyspnoea, wheezing and rhonchi must have decreased in comparison with day one of admission. A special symptoms scoring list adapted from the one used by Ojoo et al [24] is used for this (see additional file 1: appendix 1). This scoring chart scores the major exacerbation symptoms such as dyspnoea, coughing, mucus production and colour and oedema. By scoring these symptoms daily, improvement or deterioration in comparison with the previous days becomes more visual and supports the pulmonologist when applying the randomisation criteria to the patient.

Randomisation is performed on a 1:1 scale using a computer-generated randomisation list that is placed in sealed envelopes containing the allocation sequence of the two treatment groups. Randomisation is performed per participating location of the hospitals. We chose this procedure to ensure all participating hospitals have a similar proportion of patients in the intervention group and patients in the control group and that the burden for each participating home care organisations is similar. Furthermore, a block size of 6 is applied to create equal numbers in both groups.

The treatment protocol for the following four days is started after randomisation and for the intervention group the process of discharge planning is started.

### Treatment protocol day 1-3 and day 4-7

The treatment protocol during the seven days of supervised treatment can be divided in the treatment before randomisation and the treatment after randomisation.

During the first three days of the treatment all patients receive usual care. The pharmacological part of this treatment consists of systemic corticosteroids (10 days in total, first 3 days 50 mg of oral or intravenous prednisolone or other corticosteroid with an equivalent dose, following 7 days of 30 mg oral prednisolone or other corticosteroid with an equivalent dose), nebulised bronchodilators (ipratropium 500 μg/salbutamol 2.5 mg, 4-6 times per day), sub cutaneous thrombosis prophylaxis, stomach protection - because of the high dosage of corticosteroids - and, if necessary, oxygen therapy. Antibiotics are prescribed if patients meet any of the following criteria: increase of the amount of mucus, mucus purulence or CRP > 50 for which no other cause can be determined. First choice of antibiotics is co-amoxiclav. However, if previous mucus cultures show sensitivity for different antibiotics, or patients are allergic, different antibiotics can be prescribed. Antibiotics are prescribed for at least 7 days.

Non-pharmacologic usual care consists of physiotherapy for all patients and dietary advice upon indication [39]. The physiotherapist instructs the patient in breathing and coughing techniques and reactivation. A standardised (additional) written instruction was developed ensuring identical instruction in the participating hospitals. Dietary advice is indicated in case of a Body Mass Index ≤ 21 or 10% unintended weight loss in the six months prior to admission [39].

After randomisation systemic corticosteroids are continued and patients start with pressure metered dose inhaled medication via spacer (at least an β_2_-antagonist or anticolinergic with inhaled glucocorticosteroid). Patients receive inhalation instruction on the day before starting with these inhalations. Patients already using nebulised inhalation medication prior to admission, may continue this after randomisation.

The physiotherapist instructs patients to follow the written instructions at home and dietary consultation is continued as in usual care and at the dietician's judgement.

On the fourth day of admission the intervention group is transferred home and undergoes the early assisted discharge intervention. The control group remains hospitalised and receives usual care in hospital.

In both groups, patient recovery progress is monitored daily using the translated and adapted exacerbation symptom scoring chart.

### Early assisted discharge intervention

Patients randomised into the early assisted discharge group are transferred home on day four of admission. The previously described treatment is continued at home and supervised by nurses. These nurses have daily contact with the patient for four consecutive days. Main objective of the supervision of the home treatment is the observation of the patient's recovery and providing counselling and reassurance to the patient and the primary informal caregiver. The nurses also address medication compliance and inhalation techniques, provide support in applying breathing- and coughing techniques and, if applicable, provide support in adhering to dietary advices. If necessary patients, can be supported in their daily life activities (e.g. washing and dressing) by the home care organisation. During the four days of home treatment, the emphasis lies on the recovery of the exacerbation. Secondary objectives like disease management and smoking cessation are addressed during the first follow up moment one month after randomisation.

General practitioners are informed about patients' participation in the trial but are not directly involved in these patients' home care. In cases of deterioration of the patient, the patient is discussed with the treating hospital pulmonologist and if necessary the patient is readmitted to the hospital. Patients can contact the hospital 24 hours a day, 7 days a week with questions or in cases of emergency.

### Follow up visits

For both treatment groups two follow up visits at the outpatient clinic are scheduled at one month and three months after randomisation. During these visits patients are seen by their own pulmonologist and a respiratory nurse. The visits to the pulmonologist are as in usual care, the visits to the respiratory nurse have a twofold purpose. Firstly, these visits focus on the different aspects of disease management. It is at the discretion of the respiratory nurse which aspects need to be addressed in each specific patient. Secondly, these visits are used to collect, dispense and administer the questionnaires and cost diaries. Additional visits can be planned at the discretion of the pulmonologist (e.g. for additional testing) but do not fall under the study protocol. At the three month follow up visit lung function testing and a six minute walking distance test are performed as well.

### Data collection

Data are collected on five time points: on the first day of admission (T0), on the third day of admission (randomisation, T1), at the end of the supervised treatment (day 7, T2) and one month (T3) and three months after randomisation (T4).

We use self-administered questionnaires and cost diaries to obtain data. The questionnaires are administered when supervision is available. Cost diaries are supplied at two time points (T2 and T3) for the up coming period, collected at the end of each follow up period and if necessary completed under supervision.

### Effect evaluation

Table 2 provides an overview of the measures of the effect evaluation and the economic evaluation, and at which time point the measurements are performed.

**Table 2:** Overview of measurements per time point

Measurement	T0	T1	T2	T3	T4
					

Demographic characteristics	x				

Smoking	x				

Body Mass Index	x				

Living situation	x				

Comorbidity	x				

Coping style (UCL)		x			

Medical treatment prior to admission	x				

Exacerbation severity	x				

Indication for admission	x				

Clinical COPD Questionnaire (CCQ)	x	x	x		x

EuroQol 5D (EQ-5D)		x	x		x

Satisfaction					

patient satisfaction			x		x

primary caregiver satisfaction			x		x

Caregiver Strain Index			x		x

Treatment Failures			x		

Readmissions					x

Mortality					x

Cost diary			x (IG)	x	x

Discrete Choice Experiment				x	

Lung function testing					x

6 Minute Walking Distance					x

T0 = baseline; T1 = randomisation; T2 = end of supervised treatment; T3 = follow up 1; T4 = follow up 2; IG = intervention group only

#### Primary outcome measurements

Primary outcome measure in this study is the effectiveness of early assisted discharge compared to usual care expressed by the change on the Clinical COPD Questionnaire (CCQ) [38] between the third day of admission (T1) and the last day of supervised treatment (T2 = day 7). The CCQ is a disease specific, ten-item questionnaire that calculates an overall score and three domain scores: symptoms, functional state and emotional state. All items are scored on a seven point scale with 0 representing the best possible score and 6 representing the worst possible score [38]. In this study the version with a 24-hour recall period is used, reflecting the health status of the past 24 hours. The CCQ is responsive to change [38] and a study in patients admitted to the hospital with an acute exacerbation of COPD, indicated that the minimal clinical important difference (MCID) of the CCQ is 0.4 [40].

#### Secondary outcome measurements

Main secondary outcome measurement of the study is the cost-effectiveness. This will be discussed in the economic evaluation section. The following other secondary measurements will be performed. These correspond with the secondary research questions from the last paragraph of the introduction:

1. Long-term effectiveness, measured with the CCQ change over the time points from day 1 to the end of the follow-up period (T4).

2. Number of treatment failures.

3. Number of readmissions to hospital and time to readmission during the three months follow-up period.

4. Mortality and time to death during the three months follow-up.

5. Generic health related quality of life, measured with the EuroQol (EQ-5D) 5D [41]. The EQ-5D will also be used to calculate quality adjusted life year (QALYs), discussed in the economic evaluation section.

6. Effects on primary informal caregiver burden measured by the Caregiver Strain Index [42].

7. Patient and primary informal caregiver satisfaction with the program. We use a translated version of the satisfaction questionnaire used by Ojoo et al. [24] and extended it with additional questions.

#### Patient characteristics

Patients are characterised using the following variables: demographic factors (age, gender, socioeconomic status measured through level of education and income), smoking, Body Mass Index, living situation, comorbidity measured with the Charlson Comorbidity Index (CCI) [43], Coping Style measured with the Utrecht Coping List [44], medical treatment at home prior to the admission, severity of the exacerbation, indication for admission and finally severity of the disease are measured as well. In addition, severity of COPD is measured three months after admission at the end of the follow up period by performing lung function testing and a six minute walking test.

### Economic evaluation

In accordance with the broad international consensus that economic evaluations should be conducted from a societal perspective [45] this cost-effectiveness analysis will include all costs, irrespective of who actually bears them.

All direct health care and non-health care costs as well as the costs of productivity losses of patient and caregiver within the three months after randomisation will be taken into account.

The following types of resource use will be recorded to calculate direct health care costs: number and length of hospital admissions and readmissions, total amount of time of community nursing care (distinguished by nurse grade), number of visits to the emergency department, number of contacts with pulmonologist, other specialist physicians, general practitioner, respiratory nurse, dietician, physiotherapist, and social worker, number of ambulance rides and medication use. These will be recorded in cost diaries and obtained from hospital records. Costs of organisational arrangements of the early discharge scheme will also be included.

Direct non-health care costs primarily include paid and unpaid household help, including the time spent by the primary informal caregiver.

Indirect costs are costs of productivity losses. We record the days a patient is absent from paid work. We also ask informal caregivers to record the number of days off work due to caring for the patient. Costs are calculated by multiplying the volume of resource use (such as hospital days, physician visits, time spent by formal and informal caregivers) by a price per unit that includes total, not marginal costs.

In addition to the societal perspective, we will calculate the costs from the financial hospital perspective, the financial perspective of the organisation providing the home care and the perspective of the health care sector. This includes costs covered by the hospital budget, the budget of the homecare organisation and the health care sectors budget, respectively.

The principal health outcome measures in the economic evaluation are the number of patients with a clinically relevant improvement in CCQ between day of randomisation and day 7, and between day of randomisation and month 3, the change in CCQ score between day of randomisation and day 7 and day of randomisation and month 3, the number of QALYs randomisation and the end of the three-month follow-up period. The latter is calculated using the Dutch EQ-5D tariff [46].

Health outcomes will be related to cost outcomes. If one of the treatment options is more effective but also more costly, results will be presented as an incremental cost-effectiveness analysis: the additional cost per additional unit of health gain, which is calculated as the difference in mean costs between early discharge and usual inpatient hospital care divided by the in mean health effects.

### Data analysis

Data analysis will be performed according to the intention to treat principle. Data from patients who die, quit participation or are otherwise lost to follow up will be included in the analysis up until the point of drop out. Missing observations will be imputed or weighted appropriately.

All primary and secondary outcome measurements will be analysed using analysis of covariance. In order to control for dependency between the repeated measurements within one patient, and for the dependency between patients from the same hospital, multilevel analyses will be performed as well. We set the significance level at α = 0.05.

#### Primary outcome measure

The changes on the primary outcome measurement, the CCQ, will be analysed with the repeated measurements ANOVA technique. The dependent variable is the change in CCQ score from baseline (T1) to the end of the supervised treatment (T2). Treatment group is considered as independent variable and baseline CCQ score (T1) and centre of treatment are considered as covariates. Age, gender and severity of the disease will also be included in the model as covariates. If necessary, other covariates will be included in the model.

#### Secondary outcomes measurements

All time-to-event outcomes (i.e. time to readmission and time to death) will be analysed using Kaplan-Meier curves and Cox Proportional Hazards regression model. Event rates (i.e. treatment failures, readmission rates and mortality rates) will be analysed using an appropriate model for count data (e.g. poisson regression or binomial regression)

Differences in outcomes defined as the mean change from baseline (e.g. long-term effectiveness, primary informal caregiver burden, patient- and primary informal caregiver satisfaction and quality of life) will be analysed using repeated measurements ANOVA.

Patient- and caregiver preference for place of treatment will be analysed using a logistic regression model.

#### Cost-effectiveness

In order to derive the total utility experienced over the course of the investigation, the number of QALYs per patient will be calculated as the area under the utility curve.

Uncertainty around the estimates of costs and health outcomes will be addressed by bootstrapping the data with bias correction and acceleration (BCa) [47,48]. The 95% confidence interval around the difference in mean costs and health outcomes will be determined by taking the 2.5^th ^percentile and the 97.5^th ^percentile of these bootstrap replications. The bootstrap replicates will be plotted in cost-effectiveness planes (CE-planes). A CE-plane is an *x-y*-diagram with the *x*-axis representing the difference in health outcome between the treatment and usual care group and the *y*-axis representing the difference in costs. By plotting all bootstrap replicates in this diagram the uncertainty around the point estimates of the ICERs will be displayed [47]. The information from the CE-planes will be summarised into cost-effectiveness acceptability curves, which represent the likelihood that early assisted discharge is the most cost-effective option at different values of the maximum acceptable willingness to pay (WTP) for a health outcome [48].

### Sample Size calculation

Primary outcome is the change in the CCQ score between baseline (day 3 of admission) and the end of the supervised treatment (day 7).

Before the start of the study, a preliminary sample size calculation for an independent samples t-test was performed based on the results of a pilot study, where the average CCQ decreased from 3.8 on the day of admission to 2.6 by the end of the supervised treatment The standard deviation of that change was 1.04. With a MCID of 0.4, the required Cohen's effect size d would be 0.385 [99]. For a risk of a type-I error of 5% (α = 0.05) and a risk of a type-II error 20% (1-β = 0.80), the required sample size was 214.

However, primary outcome measure in this study is the change in the CCQ score from the third day of admission and the end of the supervised treatment (day 7), which is likely to have a stronger correlation with the baseline score. Therefore, a new sample size calculation for ANCOVA was performed after 85 patients had been treated, without breaking the randomisation code. Taking into account the correlation between the baseline score and the change (r = 0.288), as well as the standard deviations measured in the trial (0.988 for the intervention group and 0.922 for the control group), the required effect size f is 0.22 and the sample size is 165.

### Discrete Choice Experiment

#### Background

As part of the GO AHEAD trial we perform a discrete choice experiment (DCE) to explore the preferences of patients and their informal caregivers for different treatment arrangements. The DCE provides quantitative information on the relative importance of the characteristics of the hospital treatment and the early assisted discharge scheme and the rate at which patients are willing to trade between them.

A DCE is a type of conjoint analysis used to determine individual preferences. In this study it involves presenting respondents with a series of choices between an early assisted discharge scenario and a usual hospital care scenario. Each scenario is described in terms of several characteristics, which are called attributes.

DCEs originate from mathematical psychology and have been most widely applied in market research to determine consumer preferences for goods and services and investigate the relative importance of the characteristics of these goods and services [49,50].

#### Design

A review of literature and conversations with patients and pulmonologists have lead to the selection of seven attributes with two or three levels each for the home treatment options, while the hospital option is kept constant and is not described by attributes. The attributes for the home treatments are: type of nurse (generic or respiratory), number of home visits (1, 2 or 3 per day), copayment (€0, €50 or €100), risk of readmission to hospital within treatment period (1%, 5% or 10%), whom to contact in case of emergency (general practitioner or pulmonary ward in the hospital), number of hours of informal care (1, 3 or 5 hours per day), number of different nurses visiting the patient (1-2 or more than two).

The questionnaire consists of 14 choice scenarios, two of which have a 'right answer' and aimed at testing if the respondent understands the task. There are three versions of the questionnaire, which add up to a D-optimal design of 36 different scenarios. Each respondent is asked to complete one version of the questionnaire.

In each scenario respondents indicate a preference for one of two home treatment options or the complete hospital treatment. Respondents who initially choose the hospital option, are subsequently asked to make a choice between the two home treatments. By using this forced choice question, we ascertain that all respondents provide information about their preferences for attributes of the home treatment.

#### Data analysis

Depending on the choice pattern of the respondents, the data will be analysed using conditional logit model with alternative-specific constants, a random parameter multinomial logit model (i.e. a mixed logit model) or a multilevel latent class conditional logit model, all with and without interaction effects.

This analysis results in a regression coefficient for each level of the attributes. The estimated coefficients allow conclusions to be made about the relative importance of the attributes and possible trade-offs between them.

Furthermore, we will test whether the patients who were assigned to the early assisted discharge scheme have different preferences for various characteristics of the care delivery than patients who were assigned to the conventional inpatient hospital care.

Finally, we will test for differences in preferences between patients, their informal and formal caregivers.

## Discussion

We presented the protocol of the GO AHEAD study, which assesses the effectiveness and cost-effectiveness of an early assisted discharge intervention for patients admitted to the hospital for a COPD exacerbation. It is a multi-centre RCT comparing the early assisted discharge intervention with usual care in the hospital.

Despite research on the effectiveness of early assisted discharge for COPD exacerbations, several aspects of these hospital at home schemes remain unclear. This study will provide not only information on the effectiveness and cost-effectiveness, but also on which aspects of the intervention are important for patients to make a certain choice (DCE) and secondary outcome measurements, namely effects on quality of life, effects on primary caregiver burden and patient and primary caregiver satisfaction.

There are several critical success factors to be mentioned.

In the past two decades average length of hospital stay, for acute care of all conditions, has already decreased internationally from approximately 9 days to 6 days [51]. Average length of stay for COPD exacerbations follows these trends, with changes from approximately 8.5 days to 6.5 days [11,37,52]. Although this trend has occurred in the Netherlands as well, average length of hospital stay for COPD patients is still 10.5 days [53]. With the projected increasing prevalence of COPD, especially for women, in the following years, this leaves room for interventions that reduce length of stay.

Prolonged hospital stay is associated with the presence of comorbidities [54], continuation of conservative therapy (e.g. therapies that can only be given in hospital or pulmonologists wish to observe stable patients) [37] and complex discharge planning that requires additional home care arrangements) [37,52] among others. Moreover, comorbidities are more present in patients with more severe COPD [55], and most patients hospitalised have COPD stages III or more [52,56]. This might suggest that the Dutch hospitalised population is different from that in the United Kingdom. However, the national UK audit from 2008 [56] showed that severity of the disease of patients admitted to the hospital has not changed between 2003 and 2008 (median FEV_1 _= 38% of predicted value, GOLD stage III) and 77% of all patients admitted to hospital have one or more comorbidities.

Early assisted discharge also anticipates for the need for social work involvement during hospitalisation. In the early assisted discharge scheme care at home is arranged for a certain number of days and patients are closely monitored at home. Because of the presence of nurses at home, the possible need for prolonged or extended home care is quickly identified. Arrangements can be made more easily because patients are already in the system of the home care organisation.

In the United Kingdom, early assisted discharge for COPD exacerbations is more common in hospitals that are characterised by greater number of hospital beds, higher numbers of hospital admissions and the presence of respiratory nurses [15]. The Dutch hospitals are of similar as or larger than those in the United Kingdom [51]. The number of admissions is high (11,6 per 10.000 population in 2007 [67]) and respiratory nurses have an important role in patient care. The Dutch health care systems also has a large network of primary care organisations that deliver home care that is easily accessible for the population. Therefore the use of generic community nurses in the early assisted discharge scheme is possible.

Despite similarities between the British and Dutch health care system, organisational and financial differences between these countries exist and results from studies performed in one country, with its own characteristics, can not simply be translated to and implemented in other countries. Similarities and differences should be studied more intensively and taken into account before implementing early assisted discharge in the Dutch health care system. Possible boundary for implementation in the Netherlands are the different reimbursement systems and budgets of hospital care and home care. An integrated financing structure may facilitate the implementation in the health care system.

In this trial supervision of the treatment at home is either performed by community based, generic nurses or hospital resourced specialised respiratory nurses (nurse practitioners). The use of hospital resourced specialised respiratory nurses is the most frequently described and studied form of supervision at home. Using generic community nurses, who are more available and less costly, could enable the development of more hospital at home services. In this study both strategies for early assisted discharge (hospital resources or community based) and the different professionals involved in home care (generic nurses or specialised respiratory nurses) are being used. When sample sizes of both groups allow it, this study may provide more insight in which model for early assisted discharge is more preferable.

Compared to commonly applied measures of satisfaction, a DCE quantifies the relative importance of the characteristics and levels. It assesses the trade-offs that respondents make and provides an estimate of the overall value of early assisted discharge treatments and the usual inpatient hospital care option.

Because common satisfaction questionnaires do not quantify the relative importance of attributes and levels, it is likely that patient preferences are not represented correctly in organising the process of care delivery. This may lead to suboptimal decision-making and may impair the acceptance of early assisted discharge.

To summarise, in this contribution we presented the research protocol of a multi-centre RCT studying the effectiveness and cost-effectiveness of an early assisted discharge scheme for COPD exacerbations in the Netherlands.

## Abbreviations

BCa: Bias Correction and acceleration; Body Mass Index: body weight in kilogram/(body height in meters^2^); CCI: Charlson Comorbidity Index; CCQ: Clinical COPD Questionnaire; CE-plane: Cost-Effectiveness plane; COPD: Chronic Obstructive Pulmonary Disease; CRP: C-reactive protein; DCE: Discrete Choice Experiment; EQ-5D: EuroQol-5D; FEV_1_: Forced Expiratory Volume in one second; FVC: Forced Vital Capacity; ICER: Incremental Cost-Effectiveness Ratios; MCID: Minimal Clinical Important Difference; Pack Year (PY): (number of cigarettes smoked per day) × (number of years smoking)/20; QALY: Quality Adjusted Life Years; RCT: randomised controlled trial; WTP: willingness to pay.

## Competing interests

The authors declare that they have no competing interests.

## Authors' contributions

CU is investigator of the clinical part of the study and wrote these parts of the manuscript. LG is the investigator of the discrete choice experiment and the economic part of the study and wrote these parts of the manuscript. MV, DD, PB and JR are local investigators in the participating hospitals. MB, LE and RE are coordinators of the participating home care organisations. WL, AJ, WS, BP, AP and PL participated in the patient recruitment and follow up. FS is local investigator in one of the participating hospitals, supervisor, principal investigator and contributed to the writing of the manuscript. CP and MR are supervisors, principal investigators and grant applicators. They are the author of the study protocol and contributed to the writing of the manuscript. All authors read, edited and approved the final manuscript.

## Pre-publication history

The pre-publication history for this paper can be accessed here:

http://www.biomedcentral.com/1471-2458/10/618/prepub

## Supplementary Material

Appendix 1 **Exacerbation symptom scoring chart**. Scoring chart for exacerbation symptoms that shows the course of symptoms during the 7 days treatment. Click here for file
