# Supplementary material for: Effectiveness and cost-effectiveness of early assisted discharge for Chronic Obstructive Pulmonary Disease exacerbations: the design of a randomised controlled trial
Source: BMC Public Health. 2010 Oct 18;10:618. doi: 10.1186/1471-2458-10-618 (PMC2965725; doi:10.1186/1471-2458-10-618)
Supplement: Appendix 1 — Exacerbation symptom scoring chart. Scoring chart for exacerbation symptoms that shows the course of symptoms during the 7 days treatment. [file 1471-2458-10-618-S1.PDF]

Exacerbation symptom scoring chart

Patient number:

Date of birth:

	Date	Day 1	Day 2	Day 3	Day 4	Day 5	Day 6	Day 7
**Symptoms**								
breathlessness at rest								
breathlessness during exercise								
cough								
sputum production								
*sputum consistency**								
*sputum colour***								
oedema								
cyanosis								

+++ = severe
++ = moderate
+ = mild
- = none

* tough = 1
* mucoid =2
* none = 3

** green = 1
** brown = 2
** yellow = 3
** white = 4
** clear = 5
** none = 6
